# A Treatise on Diseases of the Air Passages: Comprising an Inquiry into the History, Pathology, Causes and Treatment of Those Affections of the Throat Called Bronchitis, Chronic Laryngitis, Clergyman’s Sore Throat, Etc., Etc.

**Published:** 1852-10

**Authors:** 


*A Treatise on Diseases of the Air Passages: comprising an inquiry into the History, Pathology, Causes, and Treatment of those Affections of the Throat called Bronchitis, Chronic Laryngitis, Clergyman's Sore Throat, etc., etc.* By HORACE GREEN, A. M., M. D., Professor of the Theory and Practice of Medicine in the New York Medical College, &c. &c. Third edition, revised and enlarged. New York. Geo. P. Putnam. 1852.

A third edition of Dr. Green's work (which has met with much favor, both at home and abroad,) having been called for, the author has carefully revised the previous issues, and added to it two new chapters on spasmodic asthma and œdema of the glottis, in both of which his favorite remedy of topical cauterization has been successfully employed. We confess we are at a loss to understand the modus operandi of cauterization of the larynx in the treatment of spasmodic asthma, unless it be by the impression made upon the nervous system of the patient, and at any rate the number of cases reported (only three) is hardly sufficient to justify a conclusion as to its general applicability. It smacks rather too much of hobby-riding, coming from one whose name is so generally associated, in this country, with the method of treatment under notice.

Throughout the whole work we notice additional matter, derived from the author's more extended experience, and calculated to increase its value. The typographical execution and "getting up" of the work are highly creditable to the publishers, who have our thanks for the copy they have kindly sent us.

